# A Traveling Scholarship

**Published:** 1892-06

**Authors:** 


### A TRAVELLING SCHOLARSHIP.

The Metropolitan Museum of Art has received a gift of $24,000 as an endowment fund, the interest of which, about $1,200, is to be awarded annually to the most proficient male student in a special class in painting in the Art School to enable him to pursue his studies abroad. The gift comes from Mrs. Amelia B. Lazarus and Miss Emilie Lazarus, the widow and daughter of the late Joseph H. Lazarus, and is in the nature of a memorial. The prize is to be known as "The Jacob A. Lazarus Travelling Scholarship." This is by far the most liberal prize yet offered to young American artists. The famous "Prize of Rome," of the School of Fine Arts in Paris is only $800.

The special class to which competition for the scholarship is open will be organized this summer by the trustees. A year's course of instruction will be pursued, and the most proficient and promising students will compete each spring for the prize, under conditions to be arranged by the trustees, the School Committee, and the instructor in charge. The winner of the prize will go abroad and study for a year or more, according to circumstances, sending home his work from time to time to indicate his progress. The class will be begun next fall.—*Ex.*

